# Supplementary material for: The broad use of the Pm8 resistance gene in wheat resulted in hypermutation of the AvrPm8 gene in the powdery mildew pathogen
Source: BMC Biol. 2023 Feb 8;21:29. doi: 10.1186/s12915-023-01513-5 (PMC9909948; doi:10.1186/s12915-023-01513-5)
Supplement: Supplementary file 4 — Additional file 4. Uncropped Western blots for Figs. 2d, e, 3c and 4b. [file 12915_2023_1513_MOESM4_ESM.pptx]

## Slide 1
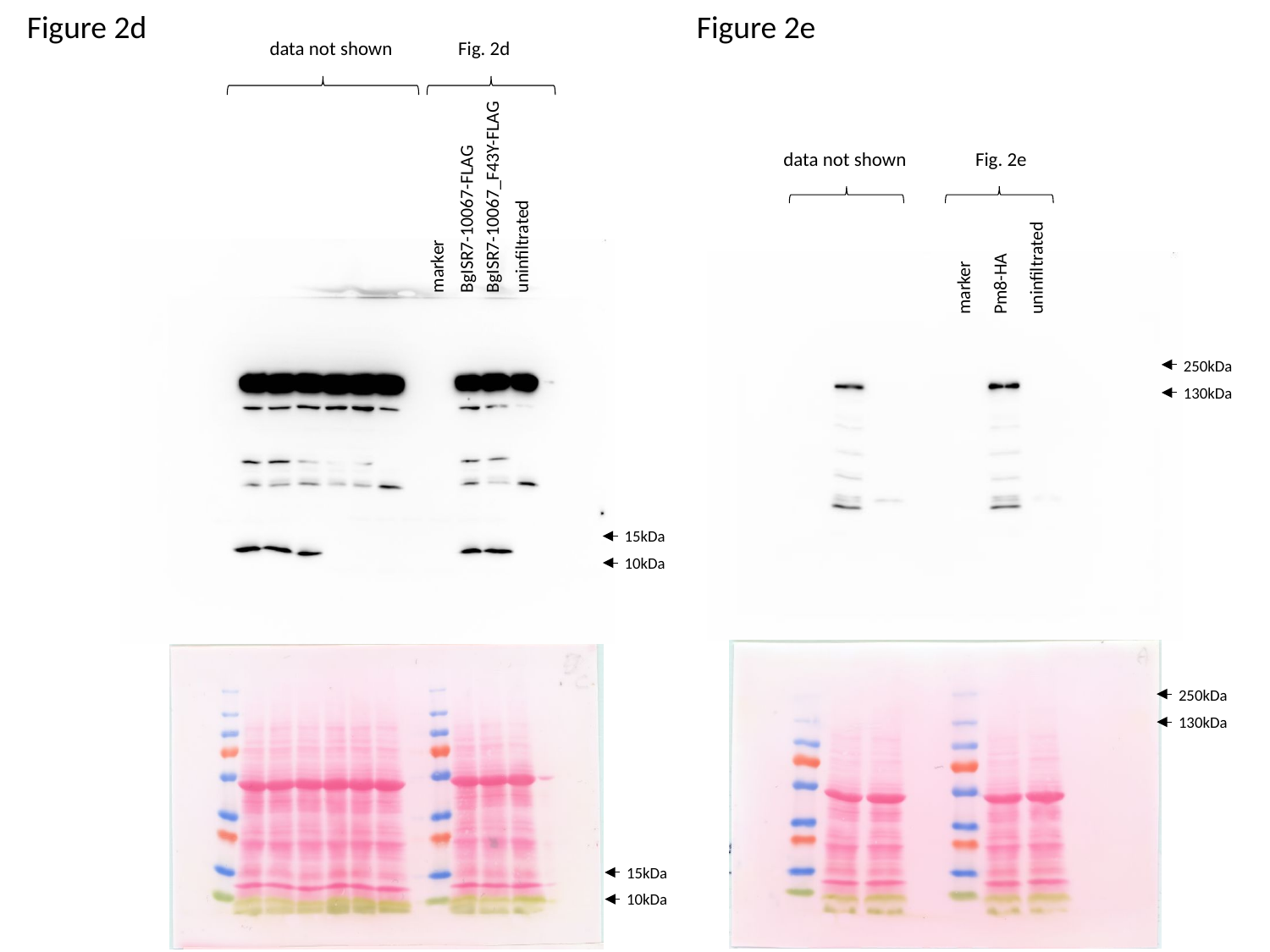

Figure 2d
Figure 2e
Fig. 2d
data not shown
data not shown
Fig. 2e
BgISR7-10067_F43Y-FLAG
BgISR7-10067-FLAG
uninfiltrated
uninfiltrated
marker
marker
Pm8-HA
250kDa
130kDa
15kDa
10kDa
250kDa
130kDa
15kDa
10kDa

## Slide 2
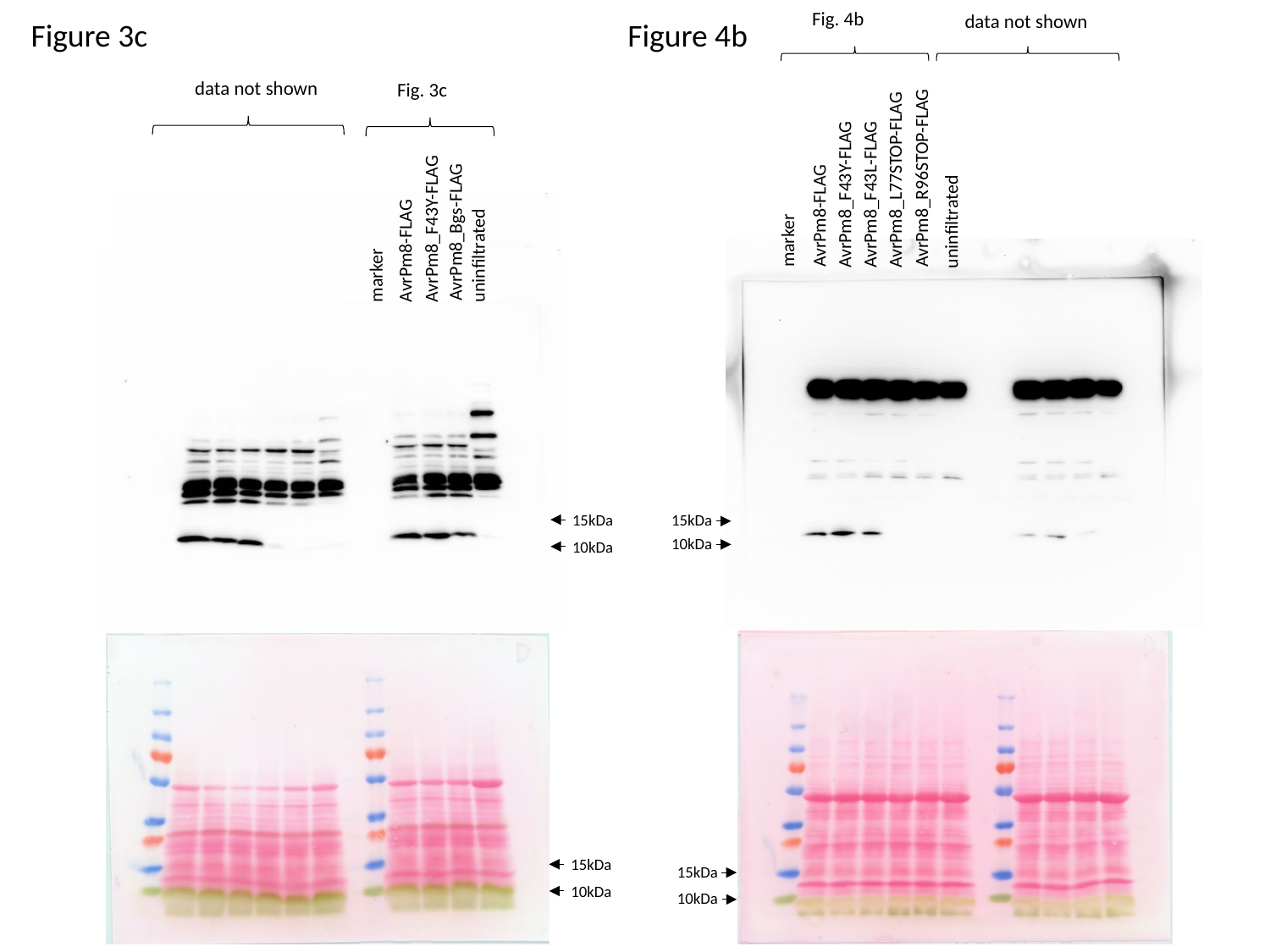

Fig. 4b
data not shown
Figure 3c
Figure 4b
data not shown
Fig. 3c
AvrPm8_L77STOP-FLAG
AvrPm8_R96STOP-FLAG
uninfiltrated
AvrPm8_F43Y-FLAG
AvrPm8_F43L-FLAG
AvrPm8_F43Y-FLAG
AvrPm8-FLAG
AvrPm8_Bgs-FLAG
marker
AvrPm8-FLAG
uninfiltrated
marker
15kDa
15kDa
10kDa
10kDa
15kDa
15kDa
10kDa
10kDa
